# Painful Memories: Reliability of Pain Intensity Recall at 3 Months in Senior Patients

**DOI:** 10.1155/2017/5983721

**Published:** 2017-02-02

**Authors:** Raoul Daoust, Marie-Josée Sirois, Jacques S. Lee, Jeffrey J. Perry, Lauren E. Griffith, Andrew Worster, Eddy Lang, Jean Paquet, Jean-Marc Chauny, Marcel Émond

**Affiliations:** ^1^Department of Emergency Medicine, Research Centre, Sacré-Coeur Hospital of Montreal, Montreal, QC, Canada; ^2^Faculté de Médecine, Université de Montréal, Montreal, QC, Canada; ^3^Axe de Recherche en Traumatologie-Urgence-Soins Intensifs du Centre de Recherche FRQ-S du CHU, Quebec City, QC, Canada; ^4^Faculté de Médecine, Université Laval, Quebec City, QC, Canada; ^5^Sunnybrook Research Institute, Toronto, ON, Canada; ^6^Department of Emergency Medicine, University of Ottawa, Clinical Epidemiology Program, Ottawa Hospital Research Institute, Ottawa, ON, Canada; ^7^Department of Clinical Epidemiology and Biostatistic, McMaster University, Hamilton, ON, Canada; ^8^University of Calgary, Calgary, AB, Canada

## Abstract

*Background.* Validity of pain recall is questioned in research.* Objective.* To evaluate the reliability of pain intensity recall for seniors in an emergency department (ED).* Methods.* This study was part of a prospective multicenter project for seniors (≥65 years old) treated in an ED for minor traumatic injury. Pain intensity (0–10 numerical rating scale) was evaluated at the initial ED visit, at one week (baseline), and 3 months. At three months, patients were asked to recall the pain intensity they had at baseline.* Results.* 482 patients were interviewed (mean age 76.6 years, SD ± 7.3) and 72.8% were female. Intraclass correlation coefficient between pain at baseline and its recall was 0.24 (95% CI: 0.14–0.33). Senior patients tended to overestimate their pain intensity by a mean of 1.2 (95% CI: 0.9–1.5) units. A stepwise multiple regression analysis showed that the variance of baseline pain recall at 3 months was explained by pain at ED visit (11%), pain at 3 months (7%), and pain at baseline (2%).* Conclusion.* The accuracy of pain intensity recall after three months is poor in seniors and seems to be influenced by the pain experienced at the time of injury.

## 1. Introduction

Pain intensity is a common outcome in pain management studies but pain intensity diaries are difficult for patients to complete reliably; therefore they are not usually used [1]. To evaluate pain treatment efficacy, researchers often rely on pain intensity recall as an estimate of pain relief [2]. In a laboratory study, recall of pain was found to be inaccurate after only a few seconds, less precise (categorical representation, i.e., light or strong pain), and more likely to be influenced by secondary information related to pain (motor response, contextual cues, etc.) [3]. However, in the clinical setting, short delays in evaluating pain recall do not seem to impair reliability. Three minutes after a painful procedure pain intensity recall largely reflects peak procedural pain and pain intensity at the end of the procedure [4]. Jensen et al. conducted a postsurgery study demonstrating that recall of pain intensity 24 hours later was a valid estimate of average pain intensity during the intervention and Perrot et al. found similar results for pain intensity recall 48 hours after musculoskeletal injections [5, 6]. Another smaller study of hospitalized orthopedic patients demonstrated similar results [7] and a very small study (16 patients) showed an accurate recall up to 5 days after the intervention [8]. Studies on pain recall after one week report contradictory results, some suggesting accurate recall [9] and others reporting significant variations in pain intensity recall [10–12]. One study found that women reported higher pain intensity at recall compared to men [13].

Beese and Morley demonstrated only fair accuracy of pain recall at two weeks postdental surgery [14]. Dunn et al. also demonstrated, at two weeks, that the combinations of pain intensity ratings were more accurate than single ratings; the mean of the recalled least, usual, and current pain intensities was closest to the daily diary ratings [15]. Pain intensity recall after more than two weeks is usually inaccurate [16–22] or minimally accurate if a three-level scale is used [23]. Consequently, the reliability of pain intensity recall over longer periods has been appropriately questioned.

Most studies define chronic pain as pain persisting for more than 3 months, so recall of pain in this setting can also be seriously questioned. Furthermore, in all these studies, seniors are poorly represented or not included. Age could be an important contributing factor since it is associated with a reduction in semantic memory for pain which can translate in a decline in reported pain [24].

The primary objective of the study was to evaluate the reliability of pain intensity recall in senior ED patients with minor trauma after a three-month follow-up period and identify factors associated with the recall of pain.

## 2. Methods

### 2.1. Study Design and Participants

This was a planned substudy of a larger prospective multicenter cohort study of functional decline experienced by seniors following a minor traumatic injury treated in an ED. Patients were recruited from April 2011 to January 2014 across seven ED teaching hospital centres of five Canadian cities (Quebec, Montreal, Ottawa, Toronto, and Hamilton). To be included, patients had to be aged 65 years or older, had to be treated in an ED within two weeks of a minor traumatic injury (lacerations, contusions, sprains, simple extremity fractures, minor thoracic injury, and mild traumatic brain injury), had to be independent in their daily living activities prior to the injury (score on ADLs of 13 or 14), had a pain intensity score of at least 1 on a 0–10 numeric rating scale (NRS) at baseline, and had to be discharged from the ED within 24 hours of arrival. Hospitalized patients, patients living in a long-term establishment, patients unable to give verbal consent, patients unavailable for follow-up, or patients unable to communicate in French or English were excluded.

### 2.2. Procedures

The study was approved by the ethics review board of each participating institution. Potential patients were recruited 24 hours a day, seven days a week by emergency physicians or research assistants. After completing a screening questionnaire to evaluate inclusion and exclusion criteria, cause of the injury, description of trauma, pain intensity assessment, loss of consciousness, and treatment plan, physicians asked the patients if they accepted to be contacted by research staff to be offered to participate in our study. After obtaining consent to participate in the study, the research staff interviewed the patients in a face-to-face meeting or by telephone within seven days of the ED visit (baseline) and then again at three and six months following the initial interview. For the present study, to insure consistency in interview quality (some measures were differently assessed by phone compared to face-to-face interviews), we selected only patients who had been contacted by phone after the ED visit (baseline) and at three-month follow-up (majority of patients). All research staff was trained on the standardized administration of the tools and questionnaires.

### 2.3. Measurements

We recorded sociodemographic variables: age, sex, race, education level, and living arrangements (alone or not). We used the Older American Resources and Service [25] scale to determine the functional status of the patients. This scale includes seven activities of daily living (ADL: eating, grooming, dressing, transferring, preparation, walking, bathing, and continence) and seven instrumental activities of daily living (IADL: meal preparation, homemaking, shopping, using transportation, using the telephone, managing medication, and managing money). Each scale ranges from 0 (dependent) to 14 (independent); patients with a score of 13 or more were considered independent. We documented the injury type and injury mechanisms and calculated a social support index taken from Quebec Health Surveys [26] using a cut-off of 60.3 as the minimum to consider adequate social support [26]. We also documented the number of prescribed medications as well as comorbidities using a list of 18 common health conditions [27]. To assess cognitive status, we used the Telephone Interview for Cognitive Status Modified (TICS-M) [28]; a cut-off of ≤31 was used to define patients with mild cognitive impairment (MCI) and a cut-off of ≤27 was used to define dementia [29].

We assessed pain intensity with a NRS ranging from 0 to 10, 0 indicating no pain and 10 indicating the worst pain imaginable. We evaluated pain intensity during the ED visit (by the triage nurse), during the initial phone interview (baseline pain intensity) and at three months. Recall of baseline pain intensity was done at three months. The three-month interview included other questions to evaluate functional decline.

### 2.4. Statistical Analyses

We used univariate statistics (Chi-square and *t*-tests) to compare the characteristics of the included and excluded patients and the intraclass correlation (ICC) to determine the agreement between the recall of baseline pain intensity (at three months) and baseline pain intensity (initial phone interview). A paired *t*-test was used to compare pain intensity at baseline and its recall at 3 months to evaluate the general direction of the difference. Mean (±95% CI) absolute pain intensity difference between pain at baseline and its recall at 3 months was also performed since there were both positive and negative differences. To better study changes in the recall of pain intensity, we divided the sample into three distinct “recall of pain intensity” groups, patients who, at recall, underestimated their pain at baseline, patients who overestimated the pain they felt at baseline, and patients who recalled correctly their pain at baseline. Bijur et al. [30] defined the minimally clinically important difference between groups as 1.3 points on 0–10 NRS in ED; however, individual patients usually select whole number so we tolerated a difference of 1 point between recall of pain and pain at baseline to assign a patient to the concordant group (e.g., a patient with a baseline pain intensity of 4/10 would need to recall a pain intensity of at least 6/10 to be classified as overestimated). Those three groups were compared on variables that could affect the recall of pain by means of one-way ANOVAs or Chi-square tests. We used post hoc Tukey-B multiple comparisons tests to compare groups of patients after a significant one-way ANOVA. Finally, we performed a stepwise multiple linear regression to find which variables best predicted the recall of pain. Because of the expected impact on the pain recall of MCI and dementia, we also performed this analysis without this group of patients. We set alpha levels at 0.05 except for data represented in Tables 1 and 2, where it was adjusted with FDR (False Discovery Rate) alpha level correction for multiple comparisons. We analyzed all data with SPSS version 22 (IBM, Somers, NY).

## 3. Results

**Figure 1:**
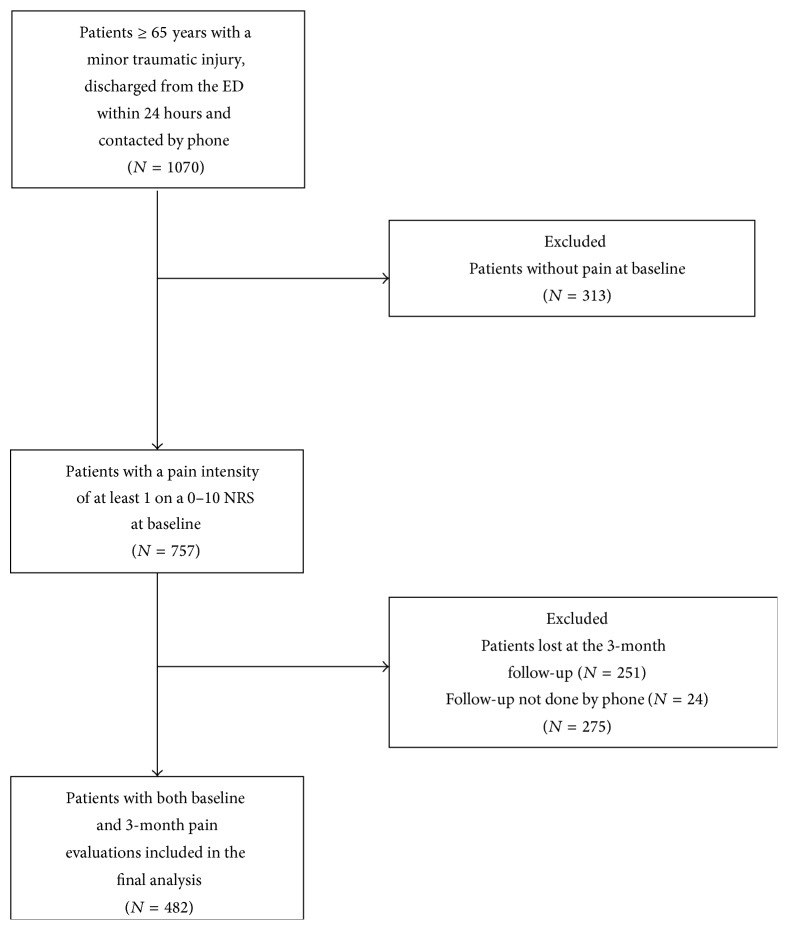
Flow chart of patient's inclusion.

At baseline (less than one week from ED visit), we interviewed 1070 patients by phone and 757 of those had pain score of at least 1 on a 0 to 10 NRS. Finally, 482 were interviewed again by phone after the three-month follow-up period (Figure 1).  Table 1 shows the characteristics of patients included in the study from our original cohort and those of the remaining 275 patients lost to follow-up or not assessed by phone (251 were lost at follow-up and 24 had face-to-face interview). Excluded patients from the original cohort were similar to included patients on all characteristics except for baseline TICS-M scores; excluded patients had a higher proportion of patients with dementia (*p* < 0.01) as per the TICS scores compared to included patients. Mean age was 76.6 years (SD ± 7.3), and a majority of patients (72.8%) were female.

The intraclass correlation coefficient between recall of pain and pain at baseline was 0.24 (95% CI: 0.14–0.33) indicating a poor agreement [31] between the two measures. At recall, patients overestimated the level of pain intensity they had at baseline by a mean of 1.2 (95% CI: 0.9–1.5) units on a 0–10 NRS (recalled pain at 3 months of 5.6 versus 4.4 at baseline; *p* < 0.001). Mean absolute pain intensity difference between pain at baseline and its recall at 3 months was 2.6 (95% CI: 2.4–2.8). Using the 1 point error tolerance (on 0–10 NRS) between baseline and recall of pain, 37.1% of patients correctly estimated their pain at baseline (1 point difference or less), 44.4% overestimated their pain (at least 2 points more), and 18.5% underestimated it (at least 2 points less).

Table 2 shows the between-group differences on variables that could affect the recall of pain. Patients who had accurate recall of pain tended to have higher medication consumption (*p* < 0.01). Patients who tended to overestimate their baseline pain at recall had significantly higher pain intensity at ED presentation than the two other groups (*p* < 0.001). No other variables including age, sex, education level, cognitive status, or pain at three months were associated with the ability to remember pain.

Results of the multiple regression analysis with recall of pain as the dependent variable and pain at ED presentation, pain at baseline, pain at three months, number of medications, age, cognitive status at three months, and education level as predictor variables are presented in Table 3. Pain recall was predicted by pain at ED presentation (explained 11% of the variance), pain at three months (explained 7% of the variance), and pain at baseline (explained 2% of the variance). Results are similar if we exclude patients with MCI and dementia.

## 4. Discussion

We demonstrated that only 37.1% of patients reliably recall their baseline pain intensity, whereas 44.4% overestimate it and 18.5% underestimate it after a three-month follow-up. We also found that pain intensity at ED presentation, pain at three months, and pain at baseline (first interview less than one week after ED visit) significantly predicted pain recall (explaining 11%, 7%, and 2% of the variance, resp.). These results are in keeping with most of the literature for a recall delay of more than one week [16–22].

As in our study, overestimated pain at recall is frequently reported in the literature [20, 21, 32, 33] and it could be explained by many factors. For example, 72.8% of our patients were female and females were shown to recall higher pain intensities than men [13, 18]. In laboratory and clinical settings, it has been established that, in a stressful context, recall of pain intensity is exaggerated [17, 21, 34] and an ED visit can certainly be viewed as a stressful event. Also, recall of pain intensity often reflects the intensity of pain at the worst and/or final part of an event. In our study, pain intensity was higher during the ED visit as compared to the baseline pain intensity of the first interview [4]. Finally, chronic pain itself (pain present for three months, like our study) is associated with overestimation of baseline pain intensity at recall [18].

Pain intensity recall has also been linked to pain intensity experienced during the time of recall, such that higher pain intensity at time of recall is associated with exaggerating baseline pain intensity and lower pain intensity at time of recall with underestimation of baseline [7, 10, 22, 33, 34]. In our study, pain at time of recall was less intense than original pain but only 18.5% underestimated their baseline pain. However, this could be explained by other factors discussed in the preceding paragraph (sex, stressful context, worst pain recall, and chronic pain).

Surprisingly, cognitive function did not influence pain recall. However, in the laboratory setting, Rainville et al. demonstrated that recalled pain ratings obtained even after very short delays are transformed into a less precise categorical format which is easier to memorize and which is possibly resistant to cognitive impairments [3].

Using only a relief scale for clinical research is also problematic, Feine et al. showed that almost all patients report relief even those whose pain had increased during the study period [22]. This also emphasizes the importance of capturing pain intensity ratings immediately in clinical research, particularly if the delay of recall is expected to be longer than one week.

Our study has some limitations that need to be considered. We lost 36% of patients at follow-up, however these patient's characteristics were similar to those of included patients (Table 1). The initial pain intensity was moderate in our study and results might be different for more intense pain. Also, we did not evaluate catastrophizing and other psychological characteristics that are known to influence recall [18, 21, 34, 35]. It is possible that patients recall the numerical ratings of pain rather than the pain they felt; however this seems unlikely after a three-month delay. Finally, the fact that patients were asked to rate current pain multiple times may also introduce additional interference on pain recall.

In conclusion, the accuracy of pain intensity is poor in senior after three months and seems mostly influenced by the pain experienced at the time of injury. The reliability of the long-term recall of pain in clinical research is thus brought into question. This emphasizes the importance of immediate assessment of pain intensity in clinical research and the need for development of tools that facilitate reliable and accurate pain recording.

## Figures and Tables

**Table 1:** Characteristics of included and excluded patients.

Characteristics	Included patients (*N* = 482)	Excluded patients (*N* = 275)	Sig.
Age (%)			
65–74	40.7	44.7	n.s.
75–84	44.6	38.9	
≥85	14.7	16.4	
Female (%)	72.8	68.6	n.s.
Ethnic group (%) Caucasian	94.3	91.4	n.s.
Education level (%)			
High school or lower	55.7	59.3	n.s.
College or higher	44.3	40.7	
Living alone (%)	38.5	35.5	n.s.
Adequate social support (%)	77.8	76.6	n.s.
Mechanism of injury (%)			
Falls own height	63.6	66.2	n.s.
Falls more than own height	16.2	12.0	
Motor vehicle accident	4.5	4.1	
Others	15.6	17.7	
Type of injury			
Mild traumatic brain injury	18.2	18.5	n.s.
Contusions	45.4	45.5	n.s.
Lacerations	22.2	25.1	n.s.
Sprains	12.9	14.5	n.s.
Fractures	30.9	28.4	n.s.
Mean (SD) number of medications	4.6 (3.8)	4.5 (3.7)	n.s.
Mean (SD) number of comorbidities	4.5 (2.6)	4.3 (2.6)	n.s.
OARS			
Baseline ADL: ≥13/14 (%)	100	100	n.s.
Baseline IADL: ≥13/14 (%)	75.9	72.0	n.s.
3-months ADL: ≥13/14 (%)	91.7	NA	
3-months IADL: ≥13/14 (%)	79.8	NA	
TICS			
Baseline MCI ≤ 31 (%)	18.2	20.1	<0.01
Baseline dementia ≤ 27 (%)	10.7	17.9	
3-months MCI ≤ 31 (%)	10.9	NA	
3-month dementia ≤ 27 (%)	8.7	NA	
Mean (SD) pain intensity at ED visit	5.5 (3.0)	5.6 (2.9)	n.s.
Mean (SD) pain intensity at baseline	4.4 (2.3)	4.8 (2.4)	n.s.
Patient with 1-pain score at baseline (%)	10.0	6.9	n.s.
Patient with 10-pain score at baseline (%)	2.3	3.6	n.s.
Mean (SD) pain intensity recall	5.6 (2.9)	NA	
Mean (SD) pain intensity at 3 months	3.2 (2.6)	NA	

Sig.: level of significance of Chi-square tests for categorical variables and of *t*-tests for continuous variables; n.s.: nonsignificant; NA: not available; OARS: Older American Resources and Service; ADL: activities of daily living; IADL: instrumental activities of daily living; TICS: telephone interview for cognitive status; MCI: mild cognitive impairment.

**Table 2:** Between-group differences on variables that could affect the recall of pain.

Characteristics	Underestimate	Correctly estimate	Overestimate	Sig.
	(*N* = 89)	(*N* = 179)	(*N* = 214)	
Mean (±SD) age	77.5 (7.2)	76.6 (7.0)	76.1 (7.5)	n.s.
Male (%)	28.4	25.7	28.0	n.s.
Education level (%)				
High school or lower	51.1	63.3	51.2	n.s.
Living alone (%)	36.4	37.6	40.2	n.s.
Adequate social support (%)	70.9	76.4	81.9	n.s.
Mean (SD) number of medications	3.6 (3.2)	5.2 (4.4)	4.6 (3.3)	<0.01^1^
Mean (SD) number of comorbidities	4.1 (2.3)	4.8 (2.8)	4.5 (2.6)	n.s.
3 months				
ADL: ≥13/14 (%)	91.0	91.1	92.5	n.s.
IADL: ≥13/14 (%)	76.1	78.8	82.2	n.s.
3 months				
TICS: MCI ≤ 31 (%)	14.3	11.2	9.2	n.s.
TICS: dementia ≤ 27 (%)	10.7	8.3	8.3	
Mean (SD) pain intensity at ED visit	4.7 (2.9)	5.0 (3.1)	6.1 (2.8)	<0.001^2^
Mean (SD) pain intensity at 3 months	2.6 (2.5)	3.5 (2.8)	3.3 (2.6)	n.s.

Sig.: level of significance of Chi-square tests for categorical variables and of one-way ANOVA for continuous variables; n.s.: nonsignificant; ADL: activities of daily living; IADL: instrumental activities of daily living; TICS: telephone interview for cognitive status; MCI: mild cognitive impairment; ^1^(Correctly estimate − Overestimate) > underestimate; ^2^Overestimate > (correctly estimate − underestimate).

**Table 3:** Results of the stepwise multiple regression analysis to predict recall of pain.

Predictors	*B* coefficient	95% CI of *B*	*R* square change	*p* value
Pain intensity at ED visit	0.31	0.21–0.40	0.11	<0.001
Pain intensity at 3 months	0.26	0.15–0.37	0.07	<0.001
Pain intensity at baseline	0.19	0.05–0.33	0.02	<0.001

Adjusted *R*^2^ = 0.20, *F* = 26.9, and *p* < 0.001. Age, education level, cognitive status at 3 months, and number of medications were not retained in the final regression model; CI: confidence interval.

## Additional Points

*Summary.* In a prospective multicenter study, senior trauma patients were contacted by phone 3 months after their injury and asked to recall the pain (on a 0–10 numerical rating scale) they had at the first interview. The accuracy of pain intensity recall was poor and seems mostly influenced by the pain experienced at the time of injury. The reliability of the long-term recall of pain in clinical research is thus brought into question. This emphasizes the importance of immediate assessment of pain intensity in clinical research and the need for development of tools that facilitate reliable and accurate pain recording.
